# Spattering mechanism of laser powder bed fusion additive manufacturing on heterogeneous surfaces

**DOI:** 10.1038/s41598-022-24828-9

**Published:** 2022-11-27

**Authors:** Toshi-Taka Ikeshoji, Makiko Yonehara, Chika Kato, Yuma Yanaga, Koki Takeshita, Hideki Kyogoku

**Affiliations:** 1 Research Institute of Fundamental Technology for Next Generation, Kindai University, K.U.RING, 1 Umenobe, Higashi Hiroshima, Hiroshima, 739-2116 Japan; 2 Technology Research Association for Future Additive Manufacturing; TRAFAM, 1-10-4 Kajicho, Chiyoda-ku, Tokyo, 101-0044 Japan; 3 Nikon Corporation, Shinagawa Intercity Tower C, 2-15-3, Konan, Minato-ku, Minato-Ku, Tokyo, 108-6290 Japan

**Keywords:** Engineering, Materials science, Optics and photonics

## Abstract

Laser powder additive manufacturing (PBF-LB) is an additive manufacturing method capable of producing high-precision and fully dense parts. However, nondestructively quality assurance of no internal defects remains challenging. Mitigating internal defects requires elucidating their formation mechanism and improving the PBF-LB process conditions. Therefore, we developed an in-situ monitoring system that combines surface morphology measurement by fringe projection and thermal field measurement with a high-speed camera. On heterogeneous surfaces in a practical multi-track PBF-LB process, a roughness index of the built part surface altered cyclically, consistent with the change in the angle between laser scanning and atmospheric gas flow. The high-speed camera monitoring showed that the melt pool was asymmetrical and spindle-shaped and that spatter was emitted mainly from the built part side of the melt pool. Furthermore, it was found that the built-part surface morphology under the powder layer affected the stability of the melt pool. As a result, a graphical representation of the melt pool and spattering for heterogeneous surfaces was proposed. Although it is still difficult to theoretically estimate the process window in which no spattering and no internal defects, in-situ monitoring equipment will provide knowledge to elucidate spattering and internal defects formation.

## Introduction

Laser powder bed fusion (PBF-LB) additive manufacturing is widely applied in the aerospace^1,2^ and medical industries^3,4^. However, the PBF-LB process has several limitations related to the deterioration of the quality of products caused by internal micro defects and the assurance of stable product manufacturing. PBF-LB process produces 3D models by compiling laser irradiated powder beds. A powder bed is a powder layer formed by a powder recoating, and then a laser irradiates to melt the powder layer to create a 2D section of the 3D model. PBF-LB requires the control of various parameters in terms of powder characteristics^5,6^, powder recoating, and building processes^7,8^. More precisely, the powder recoating conditions and the powder characteristics, including powder particle size distribution and powder flowability, affect the powder bed characteristics, e.g., uniformity of powder layer thickness, the density of powder layer, and surface roughness. Even though the building process conditions, e.g., laser irradiation and atmosphere conditions, are the same, the built material can contain internal defects when the powder bed characteristics are different. Thus, the effect of powder bed characteristics on the melting process during laser scanning is required to ensure the quality of the final products^9–12^.

Recent research on the in-situ monitoring of the powder bed and built part surface focused on clarifying the mechanism of defect formation^13–20^. Pattern projection^16^, vision sensing, and low coherence interferometry^18^ were proposed to quantify the surface morphology of the built parts^21^. However, the surface morphology of the powder bed has not been observed and sufficiently reported.

Further, extant research focused on the mechanism of defect formation during the PBF-LB process, and monitoring techniques are being developed to ensure the stable manufacturing of high-quality products^13–18^. The formation mechanism of defects caused by keyholing and spattering has been investigated using a high-speed camera^8,22–34^ and micro-synchrotron X-ray computed tomography (µSXCT)^35–41^. Observations on spattering and melt pool behavior were reported for the single laser track on a powder bed^42–50^; however, these observations do not adequately explain the practical laser scanning performed during the PBF-LB process. Most research has been conducted for a single laser track on the uniform powder bed surface. However, the practical process uses multi-tracks; each laser scans a line with a powder layer surface on one side and the solid part surface built by a previous laser scan on the other. A surface with both a powder layer and a solid part surface is called a heterogeneous surface in this research. To the authors' knowledge, there are no reports on the systematic qualification of spattering and melt pool behavior on the heterogeneous surface.

Therefore, this research aims to clarify the powder bed formation and melting process during laser scanning on a heterogeneous surface. Furthermore, an in-situ monitoring system is developed for the quality assurance of the final products manufactured using PBF-LB.

## Results

### Surface morphology and scan direction

**Figure 1:**
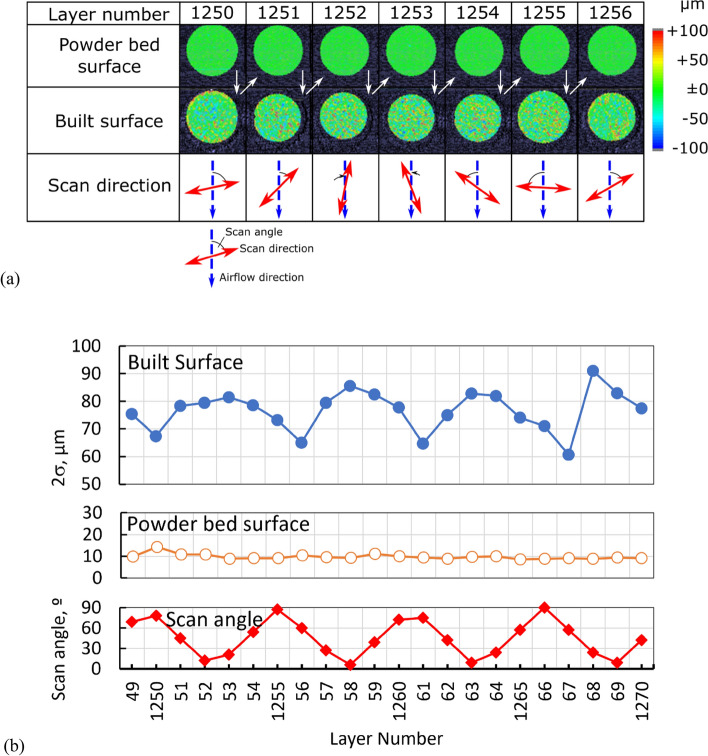
Change in monitoring images of the surface morphologies of the powder bed and the built part. (**a**) Change in the surface morphology of the powder bed and built part fabricated at the condition; $P$ = 200 W, $v$ = 665 mm/s, $h$ = 0.1 mm, $z$ = 0.05 mm, and $E$ = 30.1 J/mm^3^) from the 1250th to 1256th layer. (**b**) The values of 2σ of the powder bed and the built part from the 1249th to the 1270th layer. Scan angle is the angle to the vertical line, which is top to bottom in the surface images.

A representative surface of a specimen built under conditions for fully dense fabrication is shown in Supplementary Fig. S1. The SEM image shows a relatively regular laser beam track and deposited spatters. The image of the surface morphology measured by coherence scanning interferometry (ZYGO New View™ 9000 CSI System) indicates that the spatter height is around 100 µm. Although these images show the final surface of the specimen, the layer monitoring system records surfaces during fabrication (Fig. 1). Figure 1a shows monitoring images of the surface morphology of the powder bed and built part from the 1250th to the 1256th layer.

The powder bed was formed first in every layer, and then laser scanning fabricated the built part surface. Thus, the monitoring images of the powder bed surface were green over the surface, which indicated an almost uniform surface. However, the monitoring images of the built surface contained red and blue dots. Those scattered dots indicated the sharp peaks with + 100 µm height and valleys with −100 µm depth distributed randomly and independently. They suggested that the built part surface was roughened by laser scanning on the uniform powder bed surface.

The 2σ value of the powder bed surface was almost constant at approximately 10 µm (Fig. 1b). However, the 2σ value of the built part surface changed with the progress in layer number and varied over 60–90 µm; the peak and bottom values were observed at every sixth layer. The period of the 2σ value change of the built part coincided with the period of change in the angle between the scan direction and the atmospheric gas flow.

### Melt pool shape, process parameters, and spattering

**Figure 2:**
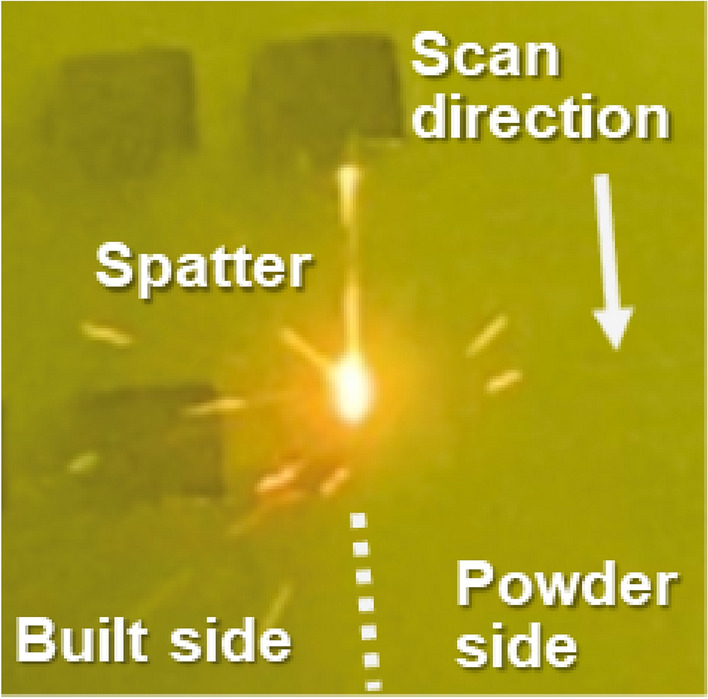
CCD camera image of laser scanning onto the powder.

Macroscopic observation of laser scanning using a CCD camera suggests the direction of spattering (Fig. 2). Under the current process conditions, spatter dispersed mainly from the built part side. In contrast, from the powder bed side, the spatter tended to emit in the vertical direction above the melt pool and was blown by the atmospheric gas flow, although the spatter did not occur much.

**Figure 3:**
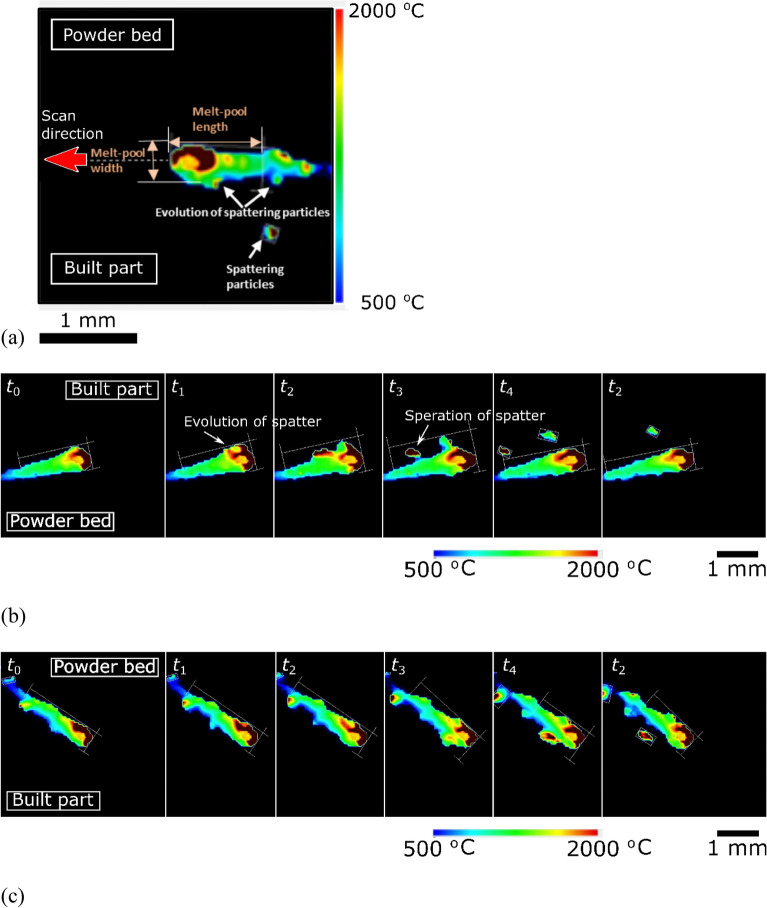
Images of a melt pool captured by the monitoring apparatus. (**a**) Representative morphology of the melt pool. (**b**) Sequential images of the melt pool behavior on a surface with a relatively lower $2\sigma$ value of the 1250th layer of the built specimen fabricated at the conditions ($P$ = 200 W, $v$ = 665 mm/s, $h$ = 0.1 mm, $z$ = 0.05 mm, and $E$ = 30.1 J/mm^3^). (**c**) Sequential images of the melt pool behavior on a relatively rough surface of the 1254th layer of the built specimen fabricated at the conditions. Time ${t}_{n}$ is $n\times 100$ ns after the first frame; $n=0$.

The high-speed melt pool monitoring system captures this tendency of spattering microscopically (Fig. 3 and Supplementary Videos 1 and 2). In the in-situ temperature field image, one side of the scan direction is the powder bed side, and the other is the built part side. The green part indicates temperatures above the liquidus temperature of Inconel 718 alloy (1336 °C); the spindle shape area represents the melt pool. The temperature of the C-shaped dark brown area at the bow of the melt pool exceeded 2000 °C. The center of the laser spot fell on the yellow and hollow center area surrounded by the dark brown area, which is considered the mouth of the keyhole.

Further, small hot particles appeared behind the tail of the melt pool and the built part side; these were the spatters.

The length and width of the melt pool were measured at the liquidus temperature of the Inconel 718 alloy (1336 °C), and the values were 400–600 µm and 1250–1600 µm, respectively.

The spatters occurred from the tip and side rim of the melt pool and then ejected from the built part side and the tail of the melt pool. The thickness of the powder bed varies locally when a built part surface with a higher $2\sigma$ value lies under a powder bed surface with a lower $2\sigma$ value. The local thickness variation can change the melt pool volume locally and rapidly change the melt pool dimensions and spattering.

### Melt pool behavior at the turning point of laser scanning

**Figure 4:**
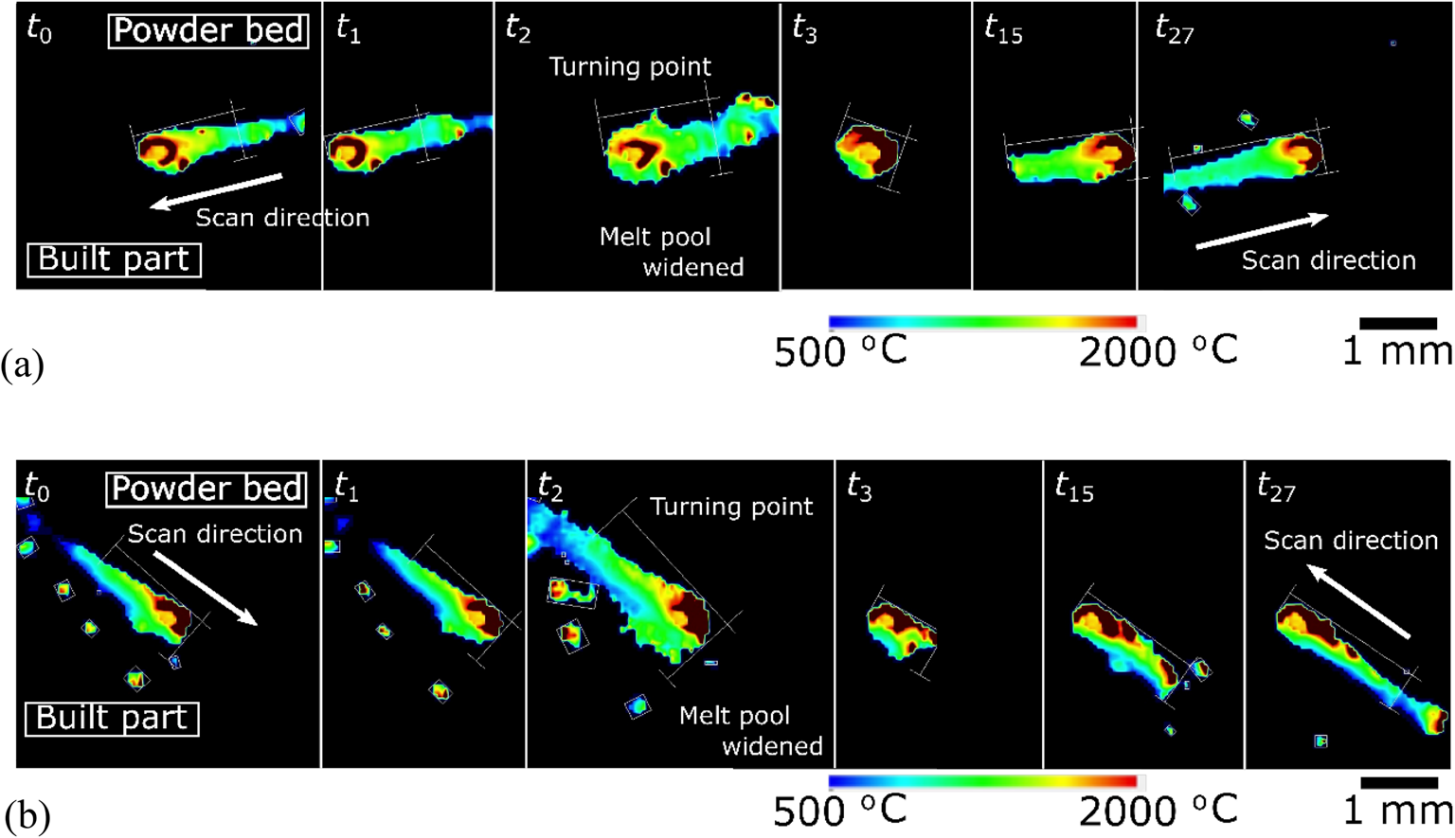
Sequential images of the melt pool behavior at the laser turning point of the (**a**) 1250th layer and (**b**) 1254th layer of the specimen fabricated at the condition ($P$ = 200 W, $v$ = 665 mm/s, $h$ = 0.1 mm, $z$ = 0.05 mm, and $E$ = 30.1 J/mm^3^). Time ${t}_{n}$ is $n\times 100$ ns after the first frame; $n=0$.

The in-situ monitoring system showed the irregularity of the melt pool at the turning point, where the laser scan direction turned 180°. This point corresponds to the edge of the parts or the edge of the domain of the scanning pattern. The time-series images of the melt pool at the turning point on the 1250th and 1254th layers are shown in Fig. 4 and Supplementary Videos 3 and 4, respectively.

A slender asymmetric melt pool travels at ${t}_{0}$ and ${t}_{1}$ and widens at the turning point, ${t}_{2}$. Then, the melt pool becomes a nearly round shape at ${t}_{3}$ and subsequently forms a slender asymmetric shape. The 1250th and 1254th layers revealed a similar tendency despite the different built surface roughnesses. The newly melted region merged with the previous melt pool formed before turning around; therefore, the width of the melt pool was almost doubled. The last melt pool cooled to solidify quickly, but the new scan still traveled a short distance, and then the melt pool became a nearly round shape. Hooper^51^ reported the enlargement of the melt pool at the turning point for Ti–6Al–4 V; the present results agree with their findings.

The heavy spattering at the turning point was also observed through the in-situ monitoring system. A large spatter was found on the built part side, and the contour shape of the melt pool was greatly disturbed, which may contained spatters and detaching melt. However, in a short time after the turning point, no spatter was emitted despite the keyhole formation because of insufficient recoil pressure development to overcome the melt's surface tension. Then, the melt pool grew in the returning scan and began to spatter again to the built part side.

**Figure 5:**
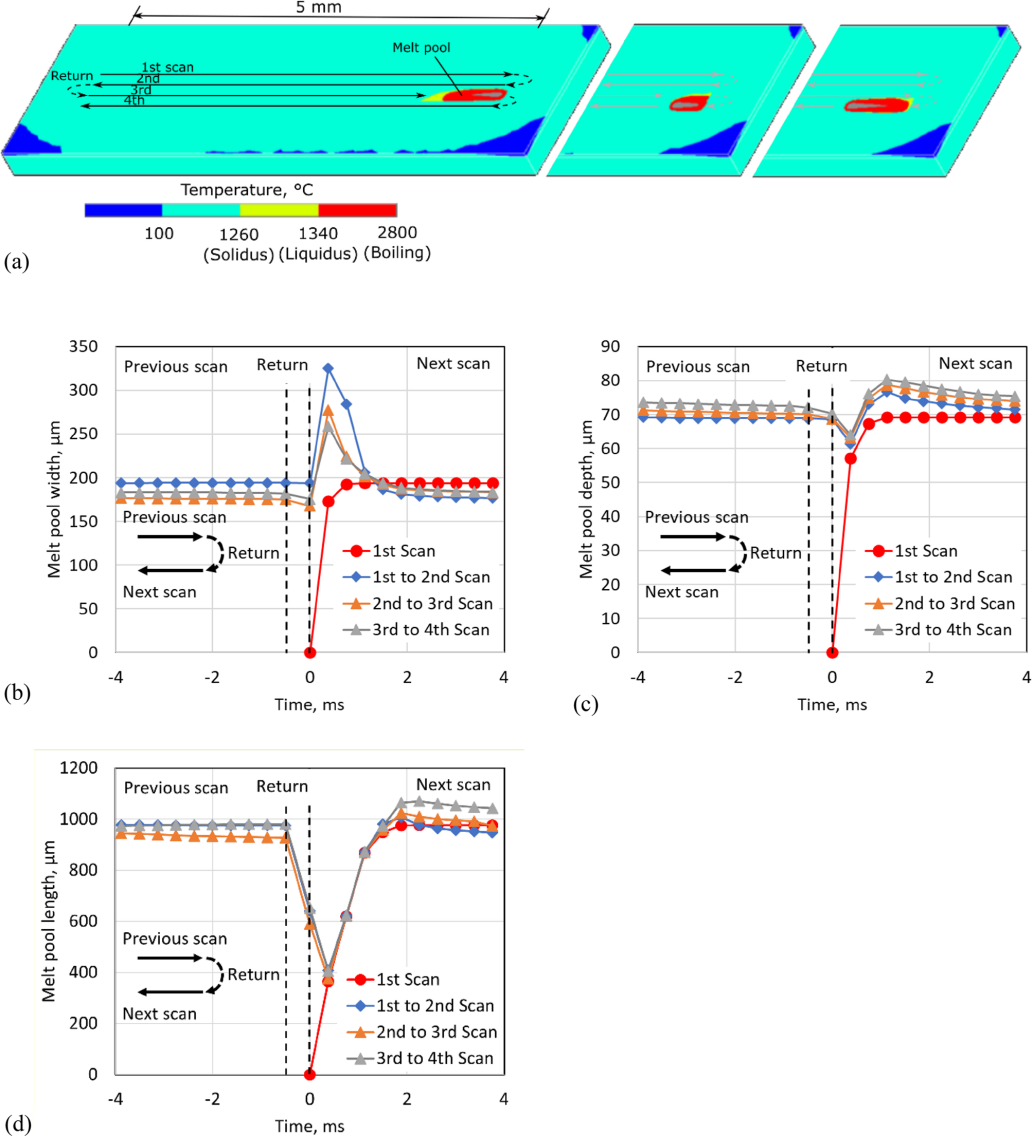
Temperature distribution of the PBF-LB process for Inconel 718. (**a**) Surface temperature distribution, (**b**) Melt pool around the turning point of laser scanning. (**c**) The depth and (**d**) width of the melt pool.

**Figure 6:**
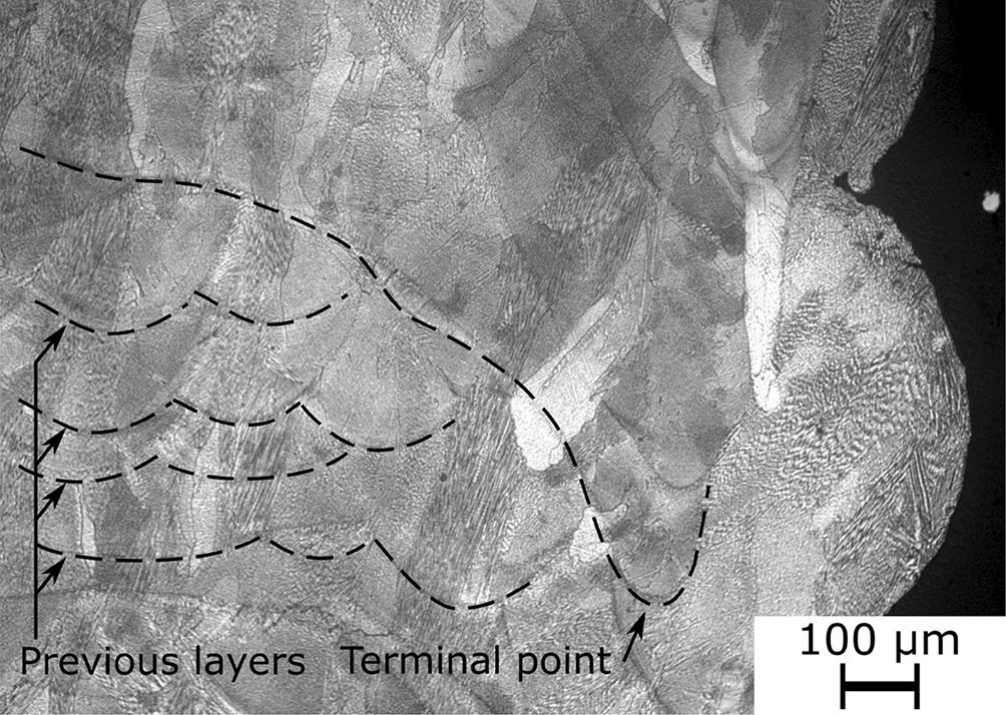
Specimen's microstructure at the edge (laser turning point).

In addition, the numerical analysis confirmed the enlargement of the melt pool. Figure 5a shows the shape change of the melt pool around the turning point between the third and fourth tracks. The estimated width of the melt pool widened by approximately 1.6 times after turning around (Fig. 5b). The melt pool depth was also deepened by about 1.2 times, although it cannot be measured via observation using the in-situ monitoring system (Fig. 5c). Even the laser irradiation once stopped at the turning point, the melt pool remained and its length was at least 400 µm (Fig. 5d). However, the numerical analysis underestimates the melt pool depth. As indicated in Fig. 6, the laser track penetrates more than three previous layers at the turning point.

Thus, the in-situ monitoring system revealed the drastic shape change in the melt pool and the heavy spattering around the turning point. A more sophisticated numerical analysis may estimate the heavy spattering around the turning point.

## Discussion

The surface morphology monitoring system reveals that every powder bed surface has a lower $2\sigma$ value. However, its subsurface, i.e., the previously built part surface, has the higher $2\sigma$ value, and the value varies by layers. Because specimens were fabricated under conditions for fully dense fabrication, few large spatters were emitted, and relatively regularly modulated beam tracks were formed. The powder recoating process buried those irregularities. Reflecting such spatters and modulation, the 2σ value of the built part surface varied over 60–90 µm. Although the machine setting of the powder bed thickness was $z$ = 50 µm, the effective powder bed thickness was estimated to be $z/\varepsilon$ = 83 µm, where the bulk density of the powder was $\varepsilon \approx$ 0.6 (Supplementary Material). The effective layer thickness was enough to bury most of the built part surface irregularity. Thus, the 2σ value of the powder bed, about 10 µm, was comparable to the difference between the maximum 2σ value of the built part, about 90 µm, and the actual layer thickness, 83 µm.

The roughness of the built part surface was not random but was introduced by the scan direction. As shown in Fig. 1b, the periodic change in the built part surface's 2σ value reflects an angle between the scan direction and the atmospheric gas flow; low for the crosswind or perpendicular gas flow and high for the head/tail wind or parallel gas flow. The crosswind cools the whole melt pool surface uniformly from the head to tail; then, the melt pool is stable and forms a surface with a relatively lower $2\sigma$ value (Supplementary Fig. S2). Conversely, the head/tail wind cools the melt pool unevenly. The headwind cools the head part first to decrease the input heat and shrink the whole melt pool, and the tailwind cools the tail part first to shorten the melt pool. Because two types of melt pools are formed in a layer for the head/tail wind case, the built part surface's $2\sigma$ value becomes higher.

The high-speed monitoring system for the melt pool behavior reveals the asymmetric shape of the melt pool on the heterogeneous surface, consisting of the powder bed on one side and the previous laser track on the other. The thermal conductivity of the powder bed is lower than that of the laser track^23,24^. This difference makes the melt pool shape asymmetric in the scan direction. In-situ monitoring in earlier research^52,53^ indicates that the melt pool shape is symmetrical because of single-track laser scanning, which means on both sides of the scan direction is the powder bed. However, our observation was for the practical scenario, wherein the powder bed and built part were on different sides. Such heterogeneous surface conditions lead to the asymmetrical melt pool.

The combination of the surface morphology monitoring data and the high-speed monitoring image suggests the relationship between the built part surface and the melt pool stability. The powder bed of the 1250th layer is formed over the built part of the 1249^th^ layer with a surface morphology of 2σ = 65 µm; the melt pool dimensions of the 1250^th^ layer are steady, and a spatter generated at the melt pool tip evolves, and is ejected (Fig. 3b). In contrast, the melt pool dimensions of the 1254th layer are larger than those of the 1250th layer and unstable (Fig. 3c). The 1254th powder bed is formed over the rough surface of the 1253rd layer's built part with 2σ = 80 µm. Spatters occur from the tip and the side rim of the melt pool and are ejected from the built part side and the tail of the melt pool. The thickness of the powder bed varies locally when a rough surface of a built part lies under the powder bed surface. The local thickness variation can change the melt pool volume locally and lead to a quick change in melt pool dimensions and spattering. Thus, the heterogeneous surface and subsurface roughness affect the melt pool dimensions; however, it is a fundamental premise that the fabrication conditions primarily influence them.

The possible measurement error must be mentioned. First, the plume emitted from the laser spot can cause a measurement error. Hooper pointed out that the effect of the hot plume emitted from the melt pool widens the observed width value^51^. Thus, Hooper's width is broader than optically measured from the specimen's cross-section. In this research, the temperature at the mouth of the keyhole was lower than its surrounding C shape area (Fig. 3). This area might be covered by the plume, whose top part was at a lower temperature. The plume hid the high-temperature inner surface of the keyhole^53^. In the case of stainless steel, the spattering and plume barely occur with a laser power of 400 W and a scanning speed of 400–500 mm/s^49^. The scanning speed in the present study, 665 mm/s, was considered higher to emit a plume; however, in-situ monitoring suggests the plume emission. Next, the limitation of the temperature range of the thermoviewer can cause a measurement error. A shorter melt pool length may be caused by the incapability of measurement at the lower temperature region corresponding to the melt pool tail because of the measurement priority in the high-temperature area.

The high-speed in-situ monitoring system revealed the asymmetric shape of the melt pool and the built-part side spattering on the heterogeneous surface, which accommodates the practical scenario but has not been reported in earlier research, to the best of the author's knowledge. The surface morphology of the built part surface and the scan direction angle to the atmospheric gas flow affect the stability of the melt pool. They also affect spattering. Thus, the fabricated material's density can be judged by monitoring the melt pool stability and the spattering. In this research, the unstabilized melt pool recovers, and it is considered due to the fully dense conditions hired. On the contrary, the melt pool will be unstable under conditions for low-density fabrication. Moreover, the combination with the laser position, the sudden melt pool instability, and the intensive spattering will lead to local defects, and the surface morphology index can report the suitability of the fabrication conditions. Since the plume emission interferes with the measurement of the melt pool dimensions and can affect the monitoring of the stability of the melt pool, it would be better to monitor the spattering.

Consider the opposite case; if a melt pool is stabilized, spattering is mitigated, a built part surface becomes a small roughness index, the fully dense fabrication conditions are achieved, and the built material is expected to be defect-free. The monitoring system developed in the present research can confirm the mitigation of spattering. The spattering and the built part surface morphology affect each other and might be equivalent for checking the suitability of the parameters; intense spattering leads to a rough built part surface and vice versa. Therefore, spattering must be mitigated to achieve conditions with fully dense fabrication.

Understanding the spattering mechanism will give a clue to mitigating spattering for the heterogeneous surface. Young et al.^39^ investigated the spattering mechanism on a homogeneous powder bed using in-situ high-speed, high-energy X-ray imaging. Furthermore, spattering is classified into five styles based on the laser power vs. scanning speed map: solid spatter, metallic jet spatter, powder agglomeration spatters, entrainment melting spatters, and defect-induced spatters. Their X-ray imaging apparatus limits the experimental configuration of the powder bed; thus, the configuration is different from that of the practical PBF-LB process in which a single laser track scans on a powder bed with a thickness of 100 µm and width of 0.5 mm. The classification of the spattering style and an explanation of the mechanism can be applied to this study even though Young et al.'s spattering mechanism is for the longitudinal direction of the melt pool. Our results may add an explanation for spattering in the crosswise direction of the melt pool.

**Figure 7:**
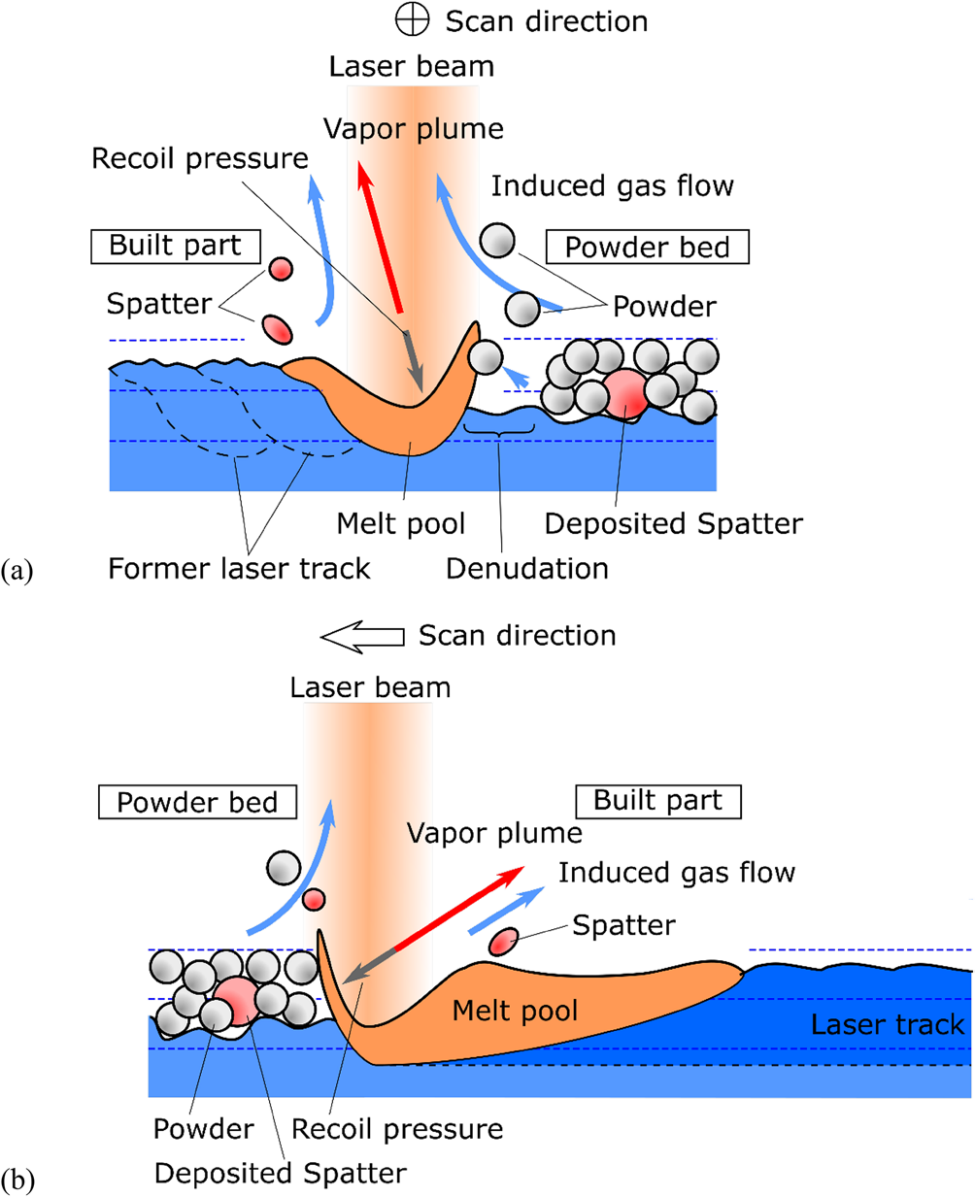
Schematic of the melt pool behavior and spatter formation. (**a**) The transverse section of the laser scan direction and (**b**) longitudinal section; melt pool length is shortened because of space limitations.

The schematics of the melt pool behavior and spatter formation are shown in Fig. 7. Laser abrasion melts a powder bed to form a melt pool, which causes metal vapor emission. The vapor pressure pushes the melt surface downwards, creating a keyhole cavity^36–39,41,52^. Above the melt surface, the vapor plume becomes a jet flow to induce an upward flow of the surrounding gas. The induced gas flow causes the powder particles adjacent to the laser tracks to denudate. The entrained powder is introduced into the melt pool and then ejected as spatters^30^. This Bernoulli effect by metal vapor jets occurs under high atmospheric pressure. When the atmospheric pressure is low enough that the atmospheric gas transport can be considered a molecular flow, the metal vapor expands locally at the laser beam spot. The expansion flow pushed the surrounding powder particle outward, resulting in denudation, as Manyalibo et al. explained with Kn number^54^. Under low atmospheric pressure, the recoil pressure is also low. Therefore, the spatter emission can be suppressed. The circulation of atmospheric gas is not enough to reduce the pressure to change the vapor jet to the expansion flow. However, it affects the direction of blowing off high-flying spatter.

The shape of the melt pool becomes asymmetric because of the higher thermal conductivity of the built part compared to the powder bed. The spattering occurs from the built part side by enlarging its melt pool. The keyhole cavity has an asymmetric cross-section; the powder bed side is a cliff-like wall, and the other side has a relatively looser slope. Thus, the plume jet from the powder bed side wall blows the melt of the built part side slope to spatter.

As indicated in Fig. 7b, the bottom surface of the powder bed in front of the bow of the melt pool is uneven, and sometimes, a relatively large spatter or ejecta is deposited. The melt volume added by the laser advance can vary to cause an irregular change in the melt pool shape. Further, the inner wall of the cavity keyhole soars nearly vertically at the bow because of the advancing laser spot. On the stern, the wall lays relatively gradually. The metal vapor jet blows from the inner bow wall to tear off the stern melt to spatter^31^. The spatter coalesces or is combined with the blown-up powders to form large ejecta, as reported by Nassar et al.^48^; the ejecta considerably affects the configuration of the melt pool. In addition, the unevenness of the surface of the built part must be considered.

The laser abrasion conditions may suppress spattering; for example, Zhen et al.^49^ reported that weak plume generation led to slight spattering on the laser scanning over the Inconel 718 powder layer with a laser power of 400 W and a scanning speed of 400–500 mm/s. Further, Yin et al.^50^ proposed a method to estimate conditions to suppress spattering for a pulse laser scan. The time at which the metal surface begins to boil after the onset of laser abrasion when a laser beam with a radius of 1/e^2^ laser spot size ${\omega }_{e}$ m and the laser power $P$ W abrades a metal plate, which is the so-called dwell time for boiling ${t}_{boiling}$, is given as

1 $$\begin{array}{*{20}c} {t_{boiling} = \frac{{\omega_{e}^{2} }}{8\alpha }\tan^{2} \left( {\frac{{\pi^{1.5} \kappa \omega_{e} T_{b} }}{{\sqrt 2 A_{p} P }}} \right), } \\ \end{array}$$

where ${T}_{b}$, $\alpha$, $\kappa$, and ${A}_{p}$ represent the boiling temperature, thermal diffusivity of the molten metal, thermal conductivity, and laser absorptivity of the plate, respectively.

In the case of the laser scan in one direction, boiling occurs when the dwell time for boiling is shorter than the time for the laser spot to pass its spot size length.

2 $$\begin{array}{*{20}c} {t_{boiling} < \frac{{\sqrt 2 \omega_{e} }}{v}.} \\ \end{array}$$

Equations (1) and (2) provide the maximum scanning speed to stop boiling, i.e.,

3 $$\begin{array}{*{20}c} {v_{boil} < \frac{8\sqrt 2 \alpha }{{\omega_{e} }}/\tan^{2} \left( {\frac{{\pi^{1.5} \kappa \omega_{e} T_{b} }}{{\sqrt 2 A_{p} P }}} \right), } \\ \end{array}$$

or the minimum pressure to boil at a scanning speed of $v$, i.e.,

3′ $$\begin{array}{*{20}c} {\frac{{\pi^{1.5} \kappa \omega_{e} T_{b} }}{{\sqrt 2 A_{p} }}/{\text{atan}}\left( {\sqrt {\frac{8\sqrt 2 \alpha }{{\omega_{e} v}}} } \right) < P_{boil} . } \\ \end{array}$$

In the same way, the substitution of the melting temperature ${T}_{m}$ instead of ${T}_{b}$ yields the boundary line of melting on the $P-v$ plot

4 $$\begin{array}{*{20}c} {v_{melt} < \frac{8\sqrt 2 \alpha }{{\omega_{e} }}/\tan^{2} \left( {\frac{{\pi^{1.5} \kappa \omega_{e} T_{m} }}{{\sqrt 2 A_{p} P }}} \right),} \\ \end{array}$$

or

4′ $$\begin{array}{*{20}c} {\frac{{\pi^{1.5} \kappa \omega_{e} T_{b} }}{{\sqrt 2 A_{p} }}/{\text{atan}}\left( {\sqrt {\frac{8\sqrt 2 \alpha }{{\omega_{e} v}}} } \right) < P_{melt} . } \\ \end{array}$$

**Figure 8:**
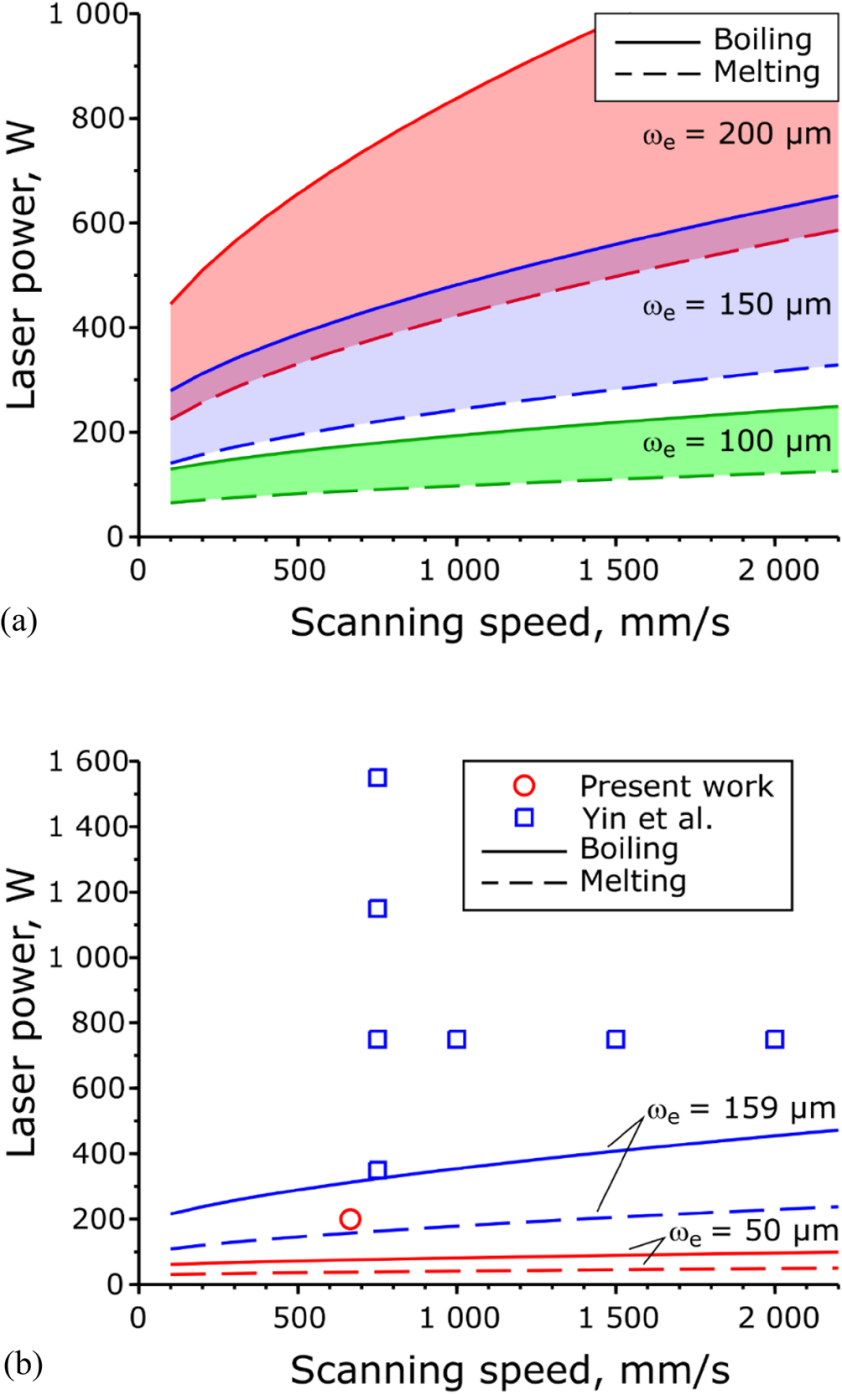
Scanning speed–laser power process map of the Inconel 718 alloy. The radius of the laser spot size is 100–200 µm. (**a**) Process window without boiling the melt pool. (**b**) Process window and fabrication condition in the experiment.

The $v-P$ plot with various ${\omega }_{e}$ is shown in Fig. 8 in the case of Inconel 718, where ${A}_{p}$ = 0.30, ${T}_{b}$ = 3190 K, $\alpha$ = 5.6 × 10^−6^ m^2^/s, and $\kappa$ = 29.6 W/m·K. The results for the 1/e^2^ spot size or $2{\omega }_{e}$ = 100–200 µm. In the process region between the solid and dashed lines, the substrate is expected to melt without boiling, which means no keyhole formation and slight spattering.

In the study of Yin et al., as the laser spot size was $2{\omega }_{e}=$ 318 µm, the spattering could be suppressed at an appropriate fabrication condition; laser power $P$ = 750 W and scanning speed $v$ = 350 m/s (Fig. 8b). The present work's conditions (the laser power $P$ = 200 W, the scanning speed $v$ = 665 mm/s) were far beyond the boiling line, and spattering was expected to inevitably occur according to Eq. (2) because the diameter of the laser spot size $2{\omega }_{e}$ is 100 µm. However, these fabrication conditions give materials with high relative density. Furthermore, the spot size of around $2{\omega }_{e}$ = 100 µm is widely used practically. This discrepancy suggests that the time to begin spattering after the onset of laser abrasion, ${t}_{spatter}$, is longer than the dwell time for boiling, ${t}_{boil}$,

$${t}_{boil}<{t}_{spatter} .$$

Equations (1) and (2) provide the necessary conditions for spattering. The ${t}_{boiling}$ value is only the dwell time for boiling; therefore, it may not spatter because of insufficient recoil pressure and the small and shallow melt pool just after the dwell time^28,31,55^. Presumably, on a $v-P$ plot, a ${P}_{spatter}\left(v\right)$ line is above the ${P}_{boil}(v)$ line,

$${P}_{boil}\left(v\right)<{P}_{spatter}\left(v\right) ,$$

and the window of no-spattering conditions will be widened. However, the corresponding Eq. (1) for ${t}_{spatter}$ is not provided so far. To obtain ${t}_{spatter}$ or ${P}_{spatter}\left(v\right)$, the criterion for keyhole dimensions to spatter is required. For example, it might be deduced by the recoil pressure described by the laser power, scanning speed, and material thermal properties. Or the in-site monitoring system will provide it based on the experiment.

In summary, for elucidating and mitigating internal defects formation of the PBF-LB process, the spattering on a heterogeneous surface, powder layer on one side, and built part surface on another were observed through a newly developed in-situ monitoring system. The in-situ measurement of surface morphology revealed that the $2\sigma$ value, which represents the surface roughness of the built part surface after laser scanning, altered cyclically with an amplitude of 60–90 μm, in agreement with the change in the angle between the laser scanning direction and the ambient gas flow. The in-situ high-speed camera monitoring showed the asymmetric melt pool and the spattering mainly to the built part side. Further, the melt pool became stable when the previously built part surface had a smaller 2σ value and conversely became unstable when the value was larger. Based on the observation, the melt pool and spattering schematics are proposed for the heterogeneous surface formed by the multi-track laser scanning during the practical PBF-LB process. Theoretical consideration to obtain the process window with no spattering conditions demands further research on keyhole formation and spattering criteria other than the boiling time estimation conducted in this research. In-situ monitoring apparatus, such as the one shown in this research, will support those research.

## Methods

### Monitoring apparatus

A PBF-LB monitoring system that can perform in-situ measurements of the surface morphology of the powder bed and the melting behavior during laser scanning was developed in this study. This system comprises the building, control, and monitoring parts (see Supplementary Fig. S3). The building part is equipped with a 1-kW single-mode Yb-fiber laser and a galvanometer laser scanner; the fiber laser wavelength is 1070 nm, and the laser beam diameter, $D$ defined by the 1/e^2^ intensity criterion is 100 µm. The capable build volume is 150 mm in diameter and 150 mm in height.

The monitoring apparatus comprises a layer monitoring system and a high-speed melt pool monitoring system. Further, the layer monitoring system is the optical surface morphology measurement system. The surface morphology is measured using the fringe projection method; it is equipped with a CCD camera (a camera with a Charge-Coupled Device image sensor) and a pattern projector. The measurement area is the entire build stage, 150 mm in diameter. The resolution is 80 µm/pixel in the horizontal x- and y- axes directions and 7 µm or less in the z-axis direction. The surface morphology is measured before and after laser scanning. In this research, the surface morphology of the powder bed is the surface shape which could be measured by the fringe pattern projection method. That means the shadow side of the particles and the too-deep hole could not be measured.

In this research, 2σ quantifies the surface morphology, representing double the standard deviation of the z-position values of the surface point group data. The point group data's z-direction position is the difference from the best-fit plane calculated by the least-squares method. 2σ value is equal to the double of root mean square height of the surface, Sq;

$$2\sigma =2{S}_{q}=2\sqrt{\frac{1}{A}\underset{A}{\overset{ }{\iint }}{z\left(x,y\right)}^{2}dxdy}$$

Further, the minimum and the maximum differences from the best-fit plane are calculated; they represent the depth and peak height, respectively.

The high-speed melt pool monitoring system can observe the temperature distribution image of the melt pool and the associated phenomena along a path coaxial to the laser beam scan. The high-speed melt pool monitoring system is equipped with a two-color thermo-viewer. The thermo-viewer consists of a high-speed camera (Photron FASTCAM SA-Z) and an optical system inserted coaxially into the laser beam for processing; thus, the center of the frame of view (FOV) coincides with the laser spot center. The FOV area is 3.98 × 3.98 mm^2^, and its sampling rate is 10 kHz; its measuring temperature range is 900–2000 °C. The temperature range is selected to improve the measurement resolution for identifying the contour of the melt pool of the Inconel 718 alloy. The liquidus temperature indicated the outline of the melt pool; 1336 °C for the Inconel 718 alloy. Further, image processing for each frame yields the width and length of the melt pool and the number and size of spatters.

Thus, the melt pool and spattering are observed, and the surface morphology of every layer is measured for the powder bed and built part.

### Fabrication conditions and evaluation methods

Round bar specimens (diameter = 10 mm and height = 100 mm) are manufactured using the gas-atomized Inconel 718 alloy powder (Carpenter Additive). These round bars are expected to machine into the JIS type 14A tensile test specimen shape specified in JIS Z2241:2011. The average powder diameter is approximately 42 µm, and the bulk density is almost $\varepsilon$ = 60%. Two thousand layers with a layer thickness of $z$ = 50 µm are compiled. The conditions include laser power $P$ = 200 W, scanning speed $v$ = 665 mm/s, hatch width $h$ = 0.1 mm, layer thickness $z$= 0.05 mm. Then the volumetric energy density is ${E}_{v}=P/vhz$ = 30.1 J/mm^3^, and areal energy density ${E}_{a}=P/vD$ = 301 J/cm^2^ where $D$ is a laser beam diameter. The scanning pattern is in a serpent form, and the scan directions are altered by 33º in every layer. Nitrogen gas flows over the build platform to reduce the oxygen content to < 0.1%. The gas flow is expected to suppress the surface oxidization and blow off the spatters and plume emitted from the melt pool surface. The monitoring system recorded the gas flow direction from top to bottom in the layer image.

The fabricated specimen has a relative density value of 100.00%. That means the fully dense material is fabricated. Fully dense material is material without significant internal defects defined in ISO/ASTM 52,900:2015. The relative density value is measured using an X-ray CT (Nikon XT H225) at a power of 100 W; the voxel size is 39 × 39 × 39 µm^3^. The measured area is the center of the round bar specimen, 10 mm in diameter and 10 mm in height. In addition, the metallurgical microstructure is optically observed after the polishing and etching of samples. Surface irregularities like fallen and adhered spatters and relatively large bumps are measured by coherence scanning interferometry (ZYGO New View™ 9000 CSI System).

The dimensional change in the melt pool is estimated via the numerical analysis of the transient heat conduction with the melting and solidifying powder and bulk metal using the finite element method using ANSYS MAPDL^31^. The analysis conditions are the conditions with fully dense fabrication mentioned above; the analysis region contains the four-tracks of laser scanning.

## Supplementary Information

Supplementary Information 1.

Supplementary Information 2.

Supplementary Information 3.

Supplementary Information 4.

Supplementary Information 5.

Supplementary Information 6.

Supplementary Video 1.

Supplementary Video 2.

Supplementary Video 3.

Supplementary Video 4.

## Data Availability

All experimental data are provided in the manuscript, or the supplementary materials are available from the corresponding author on reasonable request.
