# Supplementary material for: Spattering mechanism of laser powder bed fusion additive manufacturing on heterogeneous surfaces
Source: Sci Rep. 2022 Nov 27;12:20384. doi: 10.1038/s41598-022-24828-9 (PMC9701802; doi:10.1038/s41598-022-24828-9)
Supplement: Supplementary file 1 — Supplementary Information 1. [file 41598_2022_24828_MOESM1_ESM.docx]

**Supplementary Materials**

**Effective powder bed thickness**

The powder bed thickness at $n$-th layer, $z_{n}$, is given as

$$z_{n}=\left( 1-\varepsilon\right)z_{n-1}+z ,$$

where $\varepsilon$ and $z$ are the bulk density of the powder and the machine setting of the powder bed thickness. At the initial layer, $z_{1}=z$.

The solution to this recurrence equation is

$$z_{n}=\left( 1-{(1-\varepsilon)}^{n} \right)\cdot\frac{z}{\varepsilon} .$$

Because $0<\varepsilon<1$, its limit is

$$z_{\infty}=\frac{z}{\varepsilon} .$$

This is the effective powder bed thickness.

The layer number $n$ that $z_{n}$ can be approximate to $z_{\infty}$, i.e., that $z_{n}/z_{\infty}$ will be larger than the ratio $r$, is

$$\frac{z_{n}}{z_{\infty}}=1-\left( 1-\varepsilon\right)^{n}>r ,$$

then,

$$\frac{\log\left( 1-r \right)}{\log\left( 1-\varepsilon\right)}<n .$$

In the case of the bulk density of powder $\varepsilon=0.6$ and $r=0.999$,

$$\frac{\log\left( 1-0.999 \right)}{\log\left( 1-0.6 \right)}=7.539<n$$

Therefore, after the compilation of 8 layers, the powder bed thickness can be regarded as the effective powder bed thickness.

**Supplementary Figures**

(a)
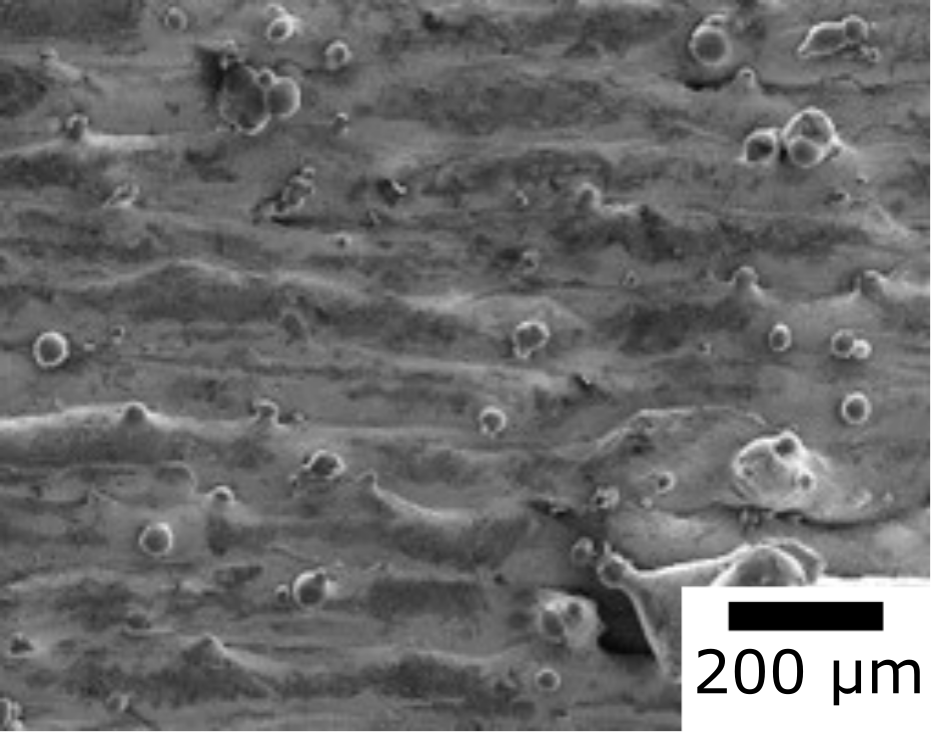


(b)

**Supplementary Figure S1**. Representative surface in (a) SEM image and (b) coherence scanning interferometry (CSI) image of the surface morphology of the built specimen fabricated at $\boldsymbol{P}$ = 200 W, $\boldsymbol{v}$ = 665 mm/s, $\boldsymbol{h}$ = 0.14 mm, $\boldsymbol{z}$ = 0.05 mm, and $\boldsymbol{E}$ = 30.1 J/mm^3^.

**
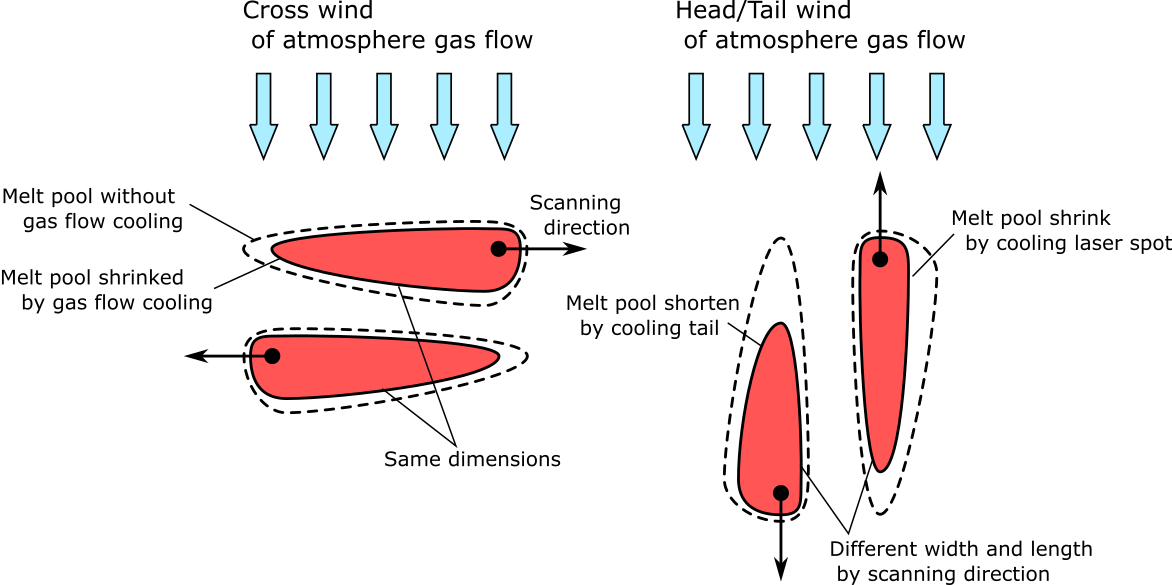
**

**Supplementary Figure S2.** Schematics of atmosphere gas flow direction on the melt pool dimensions.


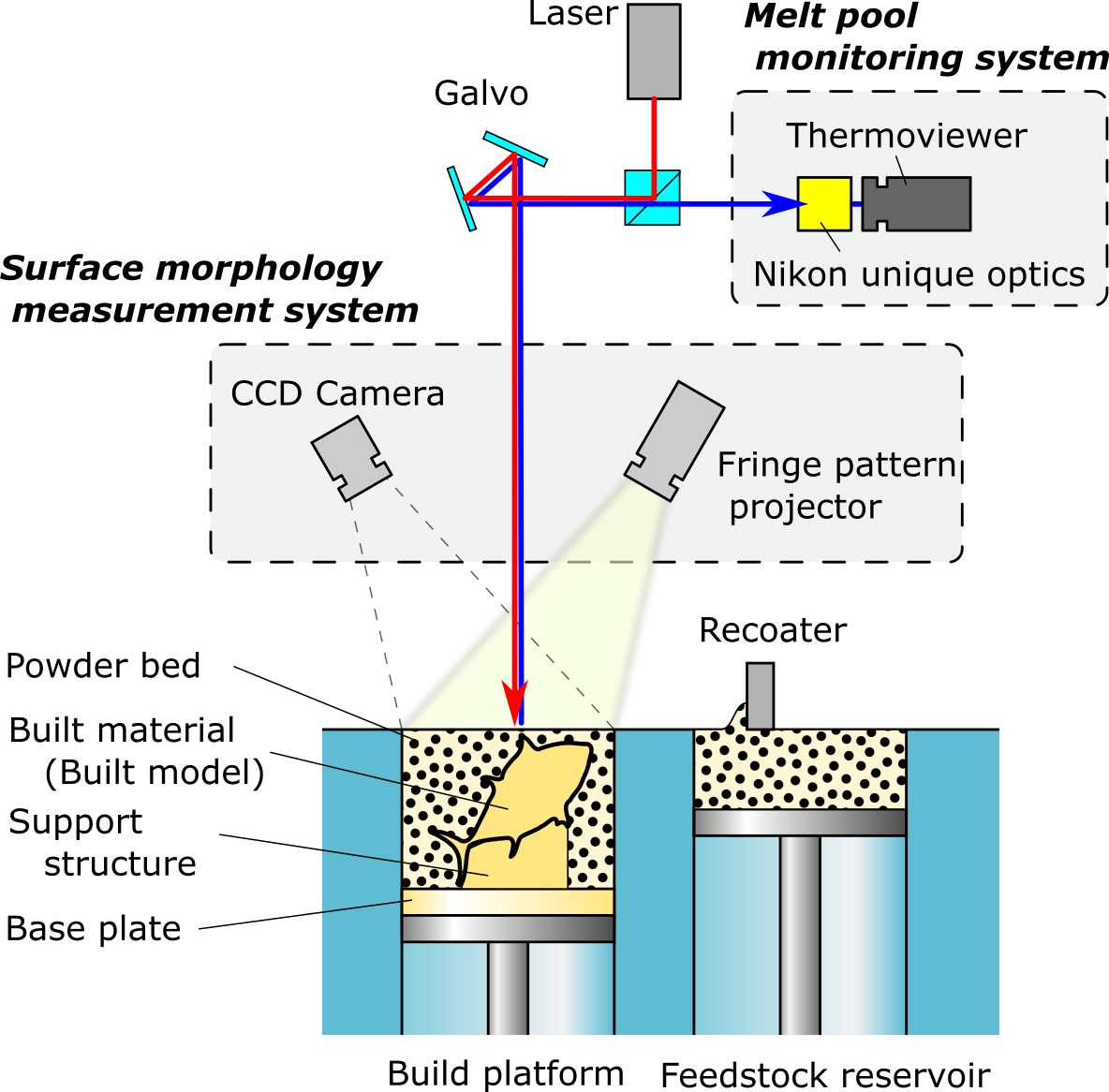


**Supplementary Figure S3.** Schematics of an in-situ monitoring system for the surface morphology of the build platform and melt pool behavior.

**Supplementary Video Legends**

**Supplementary Video 1**. Video showing the melt pool behavior on a relatively smooth surface of the 1250^th^ layer of the built specimen fabricated at $\boldsymbol{P}$ = 200 W, $\boldsymbol{v}$ = 665 mm/s, $\boldsymbol{h}$ = 0.14 mm, $\boldsymbol{z}$ = 0.05 mm, and $\boldsymbol{E}$ = 30.1 J/mm^3^.

**Supplementary Video 2**. Video showing the melt pool behavior on a relatively rough surface of the 1254^th^ layer of the built specimen fabricated at $\boldsymbol{P}$ = 200 W, $\boldsymbol{v}$ = 665 mm/s, $\boldsymbol{h}$ = 0.14 mm, $\boldsymbol{z}$ = 0.05 mm, and $\boldsymbol{E}$ = 30.1 J/mm^3^.

**Supplementary Video 3**. Video showing the melt pool behavior at the laser turning point of the 1250^th^ layer of the built specimen fabricated at $\boldsymbol{P}$ = 200 W, $\boldsymbol{v}$ = 665 mm/s, $\boldsymbol{h}$ = 0.14 mm, $\boldsymbol{z}$ = 0.05 mm, and $\boldsymbol{E}$ = 30.1 J/mm^3^.

**Supplementary Video 4**. Video showing the melt pool behavior at the laser turning point of the 1254^th^ layer of the built specimen fabricated at $\boldsymbol{P}$ = 200 W, $\boldsymbol{v}$ = 665 mm/s, $\boldsymbol{h}$ = 0.14 mm, $\boldsymbol{z}$ = 0.05 mm, and $\boldsymbol{E}$ = 30.1 J/mm^3^.
