# Supplementary figures and images for: Spattering mechanism of laser powder bed fusion additive manufacturing on heterogeneous surfaces
Source: Sci Rep. 2022 Nov 27;12:20384. doi: 10.1038/s41598-022-24828-9 (PMC9701802; doi:10.1038/s41598-022-24828-9)

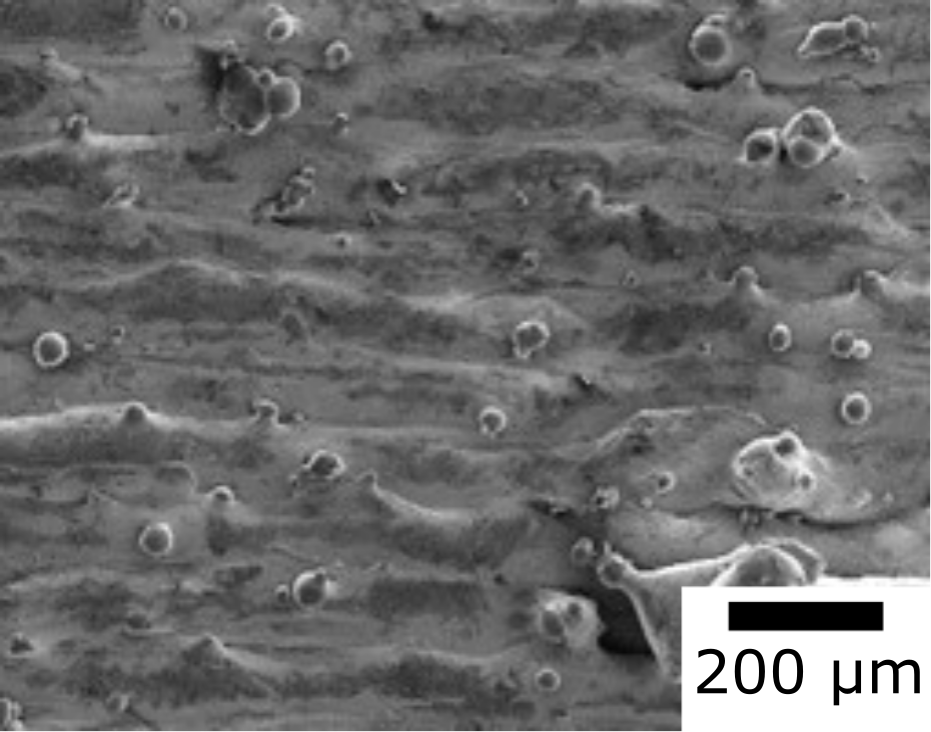

Supplement: Supplementary file 2 — Supplementary Information 2. [file 41598_2022_24828_MOESM2_ESM.png]

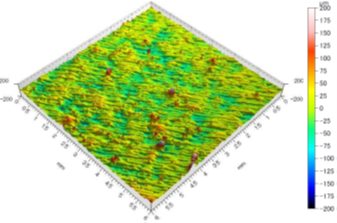

Supplement: Supplementary file 3 — Supplementary Information 3. [file 41598_2022_24828_MOESM3_ESM.png]

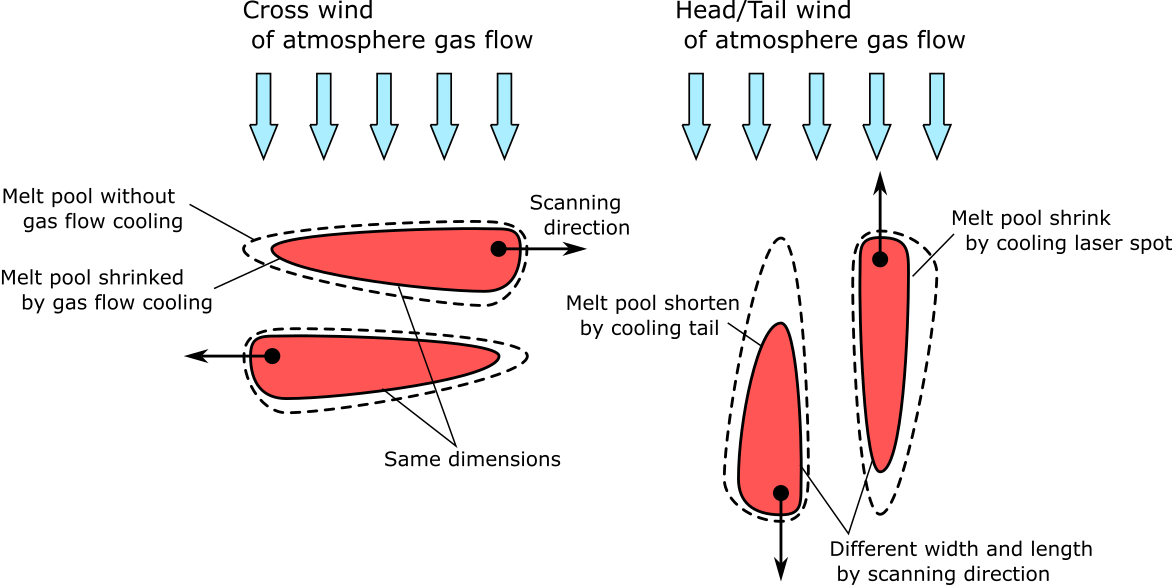

Supplement: Supplementary file 4 — Supplementary Information 4. [file 41598_2022_24828_MOESM4_ESM.png]

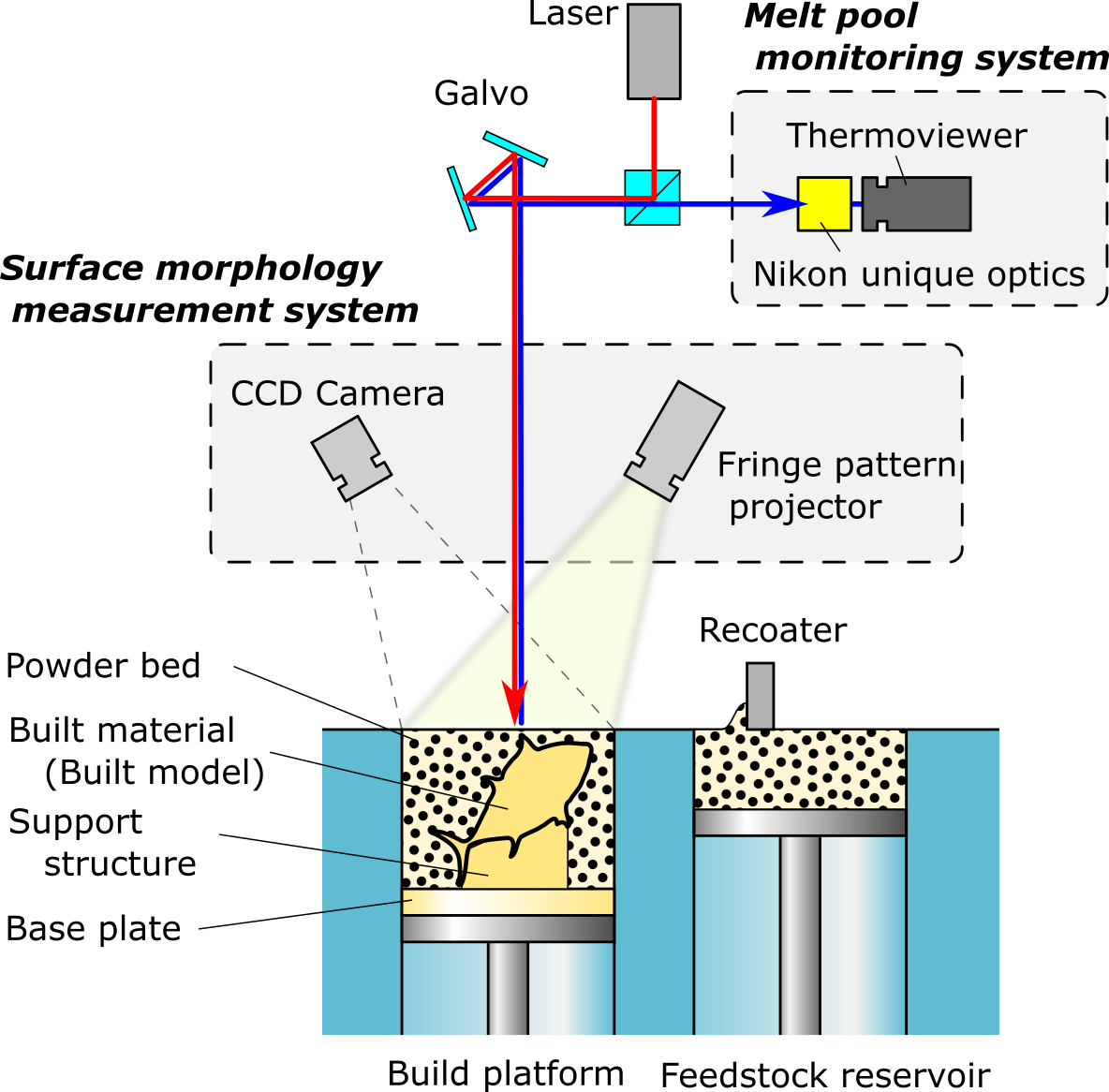

Supplement: Supplementary file 5 — Supplementary Information 5. [file 41598_2022_24828_MOESM5_ESM.png]
